# Editorial: Vector-based gene delivery in cancer immunotherapy

**DOI:** 10.3389/fimmu.2025.1587359

**Published:** 2025-03-18

**Authors:** Ning Cheng, Xiaoyue Yang, Dan Chen, Chun Xu

**Affiliations:** ^1^ State Key Laboratory of Oral & Maxillofacial Reconstruction and Regeneration, Key Laboratory of Oral Biomedicine Ministry of Education, Hubei Key Laboratory of Stomatology, School & Hospital of Stomatology, Wuhan University, Wuhan, China; ^2^ Department of Biochemical and Cellular Pharmacology, Genentech, Inc, South San Francisco, CA, United States; ^3^ Charles Perkins Centre, The University of Sydney, Camperdown, NSW, Australia; ^4^ Sydney Dental School, Faculty of Medicine and Health, The University of Sydney, Camperdown, NSW, Australia

**Keywords:** viral vector, non-viral vector, cancer immunotherapy, tumor microenvironment, gene delivery, vector engineering

## 1 Introduction

Cancer immunotherapy has revolutionized oncological treatment, establishing itself as a standard of care in the clinical setting. This transformative approach harnesses the host’s immune system to selectively target and eradicate cancer cells, offering new hope and improved outcomes for various malignancies. To achieve precise targeting of cancerous tissues and cells, gene delivery vectors such as oncolytic viral vectors and non-viral vectors are used for transferring therapeutic genetic agents, including immune checkpoint inhibitors (ICIs), pro-inflammatory cytokines, anti-angiogenic factors, and tumor suppressor genes. In addition, emerging studies have been ongoing to develop innovative strategies to reprogram the cytotoxic immune cells and the immunosuppressive tumor microenvironment (TME) (1).

In this Research Topic, “*Vector-Based Gene Delivery in Cancer Immunotherapy*”, we presented five articles that highlight cutting-edge advancements in the field. This Research Topic provides an overview of the latest progress by discussing three original research studies and two review articles published within this Research Topic. These works cover various research interests, such as vector engineering as innovative designs in cancer immunotherapy, optimization of delivery strategies through enhancing specificity and persistence, and personalized gene delivery immunotherapy.

## 2 Vector engineering: innovative designs in cancer immunotherapy

Conventional immunotherapies are limited by low patient response rates and systemic immune-related adverse events. Vector-based gene delivery has emerged as a promising strategy to overcome these challenges (1–3).

Oncolytic viral vectors exhibit dual therapeutic effects by directly lysing tumor cells and inducing anti-tumor immune activity by releasing tumor-associated antigens. For example, Yang et al. provide an innovative strategy to promote an anti-tumor response through reprogramming the immunosuppressive TME. They designed the engineered oncolytic adenoviruses oAd-SA to express SIRPα-Fc, which can block the CD47-SIRPα immune checkpoint pathway, improving macrophage phagocytosis and increasing T cell infiltration. In hepatocellular carcinoma, the commonly studied oncolytic viruses, like JX-594, induce direct tumor cell death and disrupt the tumor’s vascular system, inhibiting tumor growth and metastasis (4, 5).

Additionally, the novel gene delivery strategies targeting cancer stem cells (CSCs) are gaining attention for their potential to reduce chemoresistance, recurrence, and metastasis. In breast cancer, A et al. make use of a synthetic Notch (synNotch) receptor-engineered adenovirus (Ad-CD44-N-HIF3α4) selectively targets CD44+ CSCs in triple-negative breast cancer (TNBC), which can suppress hypoxia-induced survival signals such as VEGF and Bcl-xL, reducing TNBC aggressiveness.

Non-viral nanoparticle systems are also emerging as gene delivery vectors for tumor immunotherapy. Rakitina et al. develop the OX40L/PPT nanoparticle system which notably enhances CD8+ T-cell responses and reduces the presence of myeloid-derived suppressor cells. This system works synergistically with ICIs, improving tumor response in melanoma and colon cancer models.

## 3 Optimizing delivery strategies: enhancing specificity and persistence

The success of gene delivery in cancer immunotherapy depends largely on the method of administration and the vector used. Common delivery routes include intravenous and intratumoral delivery. Zhu et al. report that intravenous delivery can offer systemic distribution but is often challenged by immune clearance, liver sequestration, and limited tumor specificity. In contrast, Mantooth et al. review the safety and efficacy of intratumoral delivery, ensuring more localized gene expression, minimizing systemic side effects, and enhancing therapeutic precision, as proven by several clinical trials.

The choice of vector also influences the effectiveness of gene delivery-based therapies. Viral vectors, such as oncolytic adenoviruses, provide strong gene expression and potent immune activation. However, they are often limited by host immune responses, which can lead to premature clearance and toxicity. To overcome these limitations, Yang et al. report that the engineered oncolytic viral vector could selectively improve tumor targeting and bypass immune detection. Similarly, A et al. point out their engineered adenovirus ensures selective targeting of CSCs in TNBC, minimizing toxicity effects to normal tissues. Non-viral gene delivery systems, such as the OX40L/PPT nanoparticle platform in the work of Rakitina et al. show the advantage in reducing systemic toxicity compared to viral systems. However, these systems still face challenges in optimizing their physicochemical properties, such as size, charge, and surface characteristics, to improve transfection efficiency and ensure long-term therapeutic benefits.

## 4 Personalized gene delivery immunotherapy

A key advantage of vector-based gene delivery in cancer immunotherapy is the potential for personalization, which focuses on optimizing gene delivery methods and combining therapies for maximum therapeutic effect. Oncolytic viruses, such as oAd-SA engineered by Yang et al., can selectively block the CD47-SIRPα signaling axis in the TME, reprograming the immune environment to activate antitumor immune responses without causing damage to healthy tissue. A et al. construct a *synNotch* receptor fusion gene, Ad-CD44-N-HIF3α4, that triggers to release HIF-3α4 exclusively in CD44+ tumor cells, suppressing hypoxia-driven survival pathways in TNBC.

Moreover, combination therapies have shown great promise in enhancing the overall efficacy of cancer immunotherapy. For example, Rakitina et al. combine OX40L/PPT nanoparticles with ICIs and have demonstrated significant improvements in tumor control rates in preclinical models of melanoma and colon cancer. Zhu et al. summarize that the advances of OVs are combined with locoregional therapies, chemotherapy, molecular-targeted therapy, and immunotherapy to overcome limitations such as antiviral immunity and tumor resistance, demonstrating synergistic antitumor effects in preclinical and clinical studies.

## 5 Conclusion

Collectively, gene-delivery vectors offer numerous opportunities for cancer treatment. These five contributions featured in this Research Topic provide a comprehensive overview of recent advances in innovative vector engineering, optimization as safe and efficient therapeutic strategies, and transition from preclinical models to patients, which offer critical insights for overcoming treatment failure and developing effective strategies in the future.

## References

[1] Bezeljak U. Cancer gene therapy goes viral: Viral vector platforms come of age. Radiol Oncol. (2022) 56:1–13. doi: 10.2478/raon-2022-0002 35148469 PMC8884858

[2] Lundstrom K. Gene therapy cargoes based on viral vector delivery. Curr Gene Ther. (2023) 23:111–34. doi: 10.2174/1566523222666220921112753 36154608

[3] Zhang J Hu Y Yang J Li W Zhang M Wang Q. Non-viral, specifically targeted CAR-T cells achieve high safety and efficacy in B-NHL. Nature. (2022) 609:369–74. doi: 10.1038/s41586-022-05140-y PMC9452296 36045296

[4] Parato KA Breitbach CJ Le Boeuf F Wang J Storbeck C Ilkow C. The oncolytic poxvirus JX-594 selectively replicates in and destroys cancer cells driven by genetic pathways commonly activated in cancers. Mol Therapy: J Am Soc Gene Ther. (2012) 20:749–58. doi: 10.1038/mt.2011.276 PMC3321594 22186794

[5] Santry LA van Vloten JP Knapp JP Matuszewska K McAusland TM Minott JA. Tumour vasculature: Friend or foe of oncolytic viruses? Cytokine Growth Factor Rev. (2020) 56:69–82. doi: 10.1016/j.cytogfr.2020.07.007 32893095

